# Sixty years of European Renal Association (ERA) Registry data on kidney disease: visualizing differences in clinical practice

**DOI:** 10.1093/ckj/sfae120

**Published:** 2024-05-23

**Authors:** Vianda S Stel, Kitty J Jager, Alberto Ortiz

**Affiliations:** ERA Registry, Department of Medical Informatics, Amsterdam UMC, University of Amsterdam, Amsterdam, The Netherlands; Amsterdam Public Health Research Institute, Quality of Care, Amsterdam, The Netherlands; ERA Registry, Department of Medical Informatics, Amsterdam UMC, University of Amsterdam, Amsterdam, The Netherlands; Amsterdam Public Health Research Institute, Quality of Care, Amsterdam, The Netherlands; Department of Nephrology and Hypertension, IIS-Fundacion Jimenez Diaz UAM, Madrid, Spain; Department of Medicine, Universidad Autonoma de Madrid, Madrid, Spain

Sixty years ago, in 1964, a European Registry was established with data on patients with kidney failure who were treated with kidney replacement therapy (KRT). An initial report was published in 1965 on 271 patients starting hemodialysis and 6 patients starting peritoneal dialysis with a 1-year mortality of 40%–50% [1]. In addition, it reported on 258 kidney transplant operations that had been performed in Europe [1]. In the early 1970s, the Registry moved to Munich, Germany, and both dialysis and kidney transplant patients were brought together in a single database that allowed tracking of patients’ treatment sequence [2]. In 1976, the Registry moved to London, UK, and after a successful period, the Registry could not cope with the rapidly increasing amount of data they received from about 4000 renal centers. Therefore, the Registry moved to its current location in Amsterdam, the Netherlands, in 2000, and instead of collecting data from renal centers, the Registry started collecting data through national and regional renal registries [2]. Nowadays, the Registry collects data on more than half a million patients with kidney failure receiving KRT. In this editorial we will focus on the current activities and data of the European Renal Association (ERA) Registry (formerly named the ERA-EDTA Registry) and discuss key findings highlighting the main value of renal registries.

To celebrate the 60th anniversary of the ERA Registry, a special collection of articles contains 20 ERA Registry papers published in *NDT* or *CKJ* that are still of value today, which will be discussed in more detail below. Besides conducting research, the ERA Registry is active in teaching nephrologists and researchers about epidemiology, mainly in the nephrology context. The first introductory course on epidemiology took place in 2004, and since then 35 courses have taken place across Europe. The ERA Registry has also been writing articles describing basic and advanced research methodology since 2007 in collaboration with colleagues from Reggio (Italy) and Leiden (the Netherlands). Moreover, the ERA Registry hosts research fellows to contribute to their training in epidemiology, data analyses and writing scientific articles. A last important activity of the ERA Registry is assisting in coding and definitions, such as in the ERA primary renal disease (PRD) codes, ERA causes of death (COD) codes and treatment modality codes. The ERA Registry plays a key role in ensuring that countries collect these data and code them in a similar way, and in providing updates in coding systems when needed.

**Figure 1:**
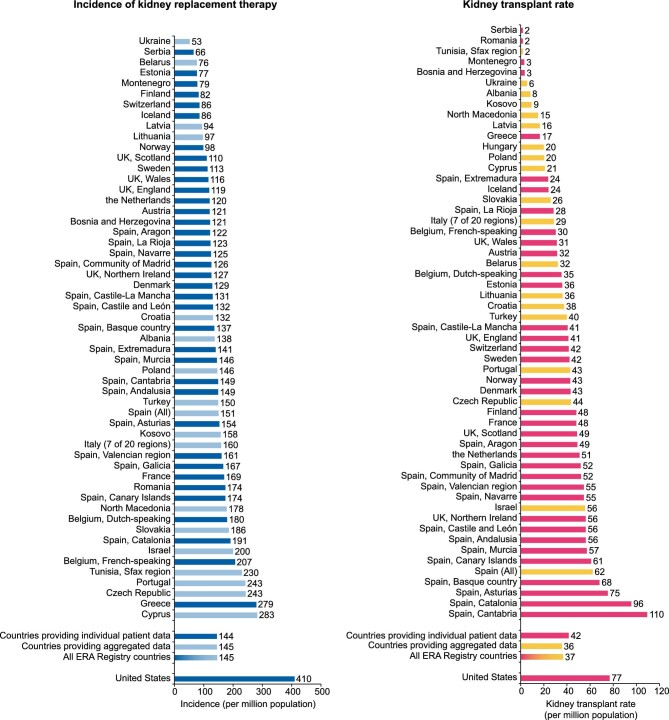
Unadjusted incidence of kidney replacement therapy (left panel) and unadjusted kidney transplants (right panel) pmp by country or region, and for Europe as a whole and the USA. Registries providing individual patient data are shown as dark bars, and registries providing aggregated data as light bars. As Hungary KRT incidence data were not available in 2021, data on Hungary are only included in the kidney transplant figure.

The ERA Registry data is a precious asset. In 2023, 54 national and regional renal registries from 36 countries in Europe or bordering Europe and the Mediterranean Sea provided data on KRT patients to the Registry office. Participating registries typically have 100% coverage of their general population and for more than 60% of the participating registries it is possible to follow individual patients over time. The ERA Registry is currently setting up a European Chronic Kidney Disease (CKD) stage 4/5 Registry in which patients will be linked to the ERA Registry once they start KRT. In addition, the ESPN/ERA Registry collects variables on KRT in approximately 28 000 children from almost every European country. An effort is currently underway to establish a European CKD 4/5 Registry in children and to link this dataset to the ESPN/ERA Registry and ERA Registry, which would allow the monitoring of disease progression in childhood and adulthood [3]. Finally, the EQUAL study which is coordinated by ERA Registry staff, includes clinical data from six European countries where elderly people with advanced kidney disease are followed once their estimated glomerular filtration rate falls below 20 mL/min.

Data from renal registries are essential in characterizing the burden of KRT and also in identifying significant disparities in the number of patients on KRT and their outcomes across individual countries. To this end, renal registry data may identify or generate hypotheses about underlying clinical problems, which may trigger a cycle of quality improvement. This means that renal registries are essential not only for healthcare planning but also for benchmarking and quality improvement, and could therefore play an important role in reducing inequalities and optimizing kidney care.

This added value of renal registries will also be illustrated by the selected ERA Registry articles in the special collection of *NDT* and *CKJ*. This collection contains the latest summaries of the ERA Registry annual report [4, 5] as well as other scientific publications based on the ERA Registry database [6–12], the ESPN/ERA Registry database [3, 13], the EQUAL database [14, 15] and other data collections [16–19]. In addition, the special collection includes several highly cited methodological papers because we believe they belong in the compilation alongside the Registry's expertise [20–22].

The two summary articles of the ERA Registry annual report provide the latest information about the disparities of the epidemiology of KRT within Europe [4] and between Europe (across countries providing individual patient data) and the USA [5]. In 2021, the unadjusted KRT incidence varied >5-fold, ranging from 53 per million population (pmp) in Ukraine to 279 pmp in Greece (Fig. 1, left panel) [4]. In the same year, the KRT incidence in the USA (410 pmp) was three times higher than that in Europe (144 pmp) (Fig. 1, left panel) [5]. Moreover, the number of kidney transplants performed pmp varied widely between individual countries, with the highest kidney transplant rates found in Cantabria in Spain (110 pmp) (Fig. 1, right panel) [4]. Of interest, despite the much higher kidney transplantation rate in the USA (77 pmp) than in Europe (42 pmp) (Fig. 1, right panel) [5], approximately half of all European prevalent patients were living with a functioning graft, while in the USA this was only one-third [4]. Great examples exist where renal registries play an important role in optimizing kidney care. One such example comes from Finland, where after noticing that their preemptive kidney transplant rate was the lowest in Europe, they took measures and increased their preemptive kidney transplant rate [4].

Several other ERA Registry studies show disparities across countries and/or changes in trends over time on for example the incidence of KRT [6], kidney transplant rates [7], characteristics of first kidney transplant patients [8], and trends in KRT survival [6]. Other studies focused on patient subgroups [9, 10]. For example, the standardized incidence of KRT for kidney failure due to primary glomerular diseases (PGD) was 2.3-fold different across European countries, while 7.5%–76.1% of PGDs were not supported by histology, raising questions about the cause of CKD [9]. Patients on extended-hours (≥6 h/treatment) hemodialysis had a 27% lower risk of death compared with patients treated for 3.5–4 h/treatment [10]. Another ERA Registry article reported on the lifetime risk on KRT which ranged from 0.44% to 2.05% at 20 years and from 0.17% to 1.59% at 70 years across countries, and was twice as high in men as in women [11].

Many ERA Registry articles aimed at understanding the reasons for the differences in the epidemiology of KRT. For example, the study on trends in kidney transplant rates [7] was combined with information on potentially successful measures and perceived barriers that may guide in determining strategies to increase kidney transplant rates [16]. Another study aimed at understanding the decrease in the incidence of KRT in elderly patients in the Netherlands in comparison with other European countries [12]. The findings may partly reflect the different policies regarding the acceptance of elderly patients to KRT but also differences in willingness of older patients to undergo dialysis. In other ERA Registry studies nephrologists and patients were surveyed about (involvement in) treatment modality choice [17, 18]. In line with the abovementioned studies, also the ESPN/ERA Registry data revealed substantial disparities in the epidemiology of children on KRT and their outcomes across Europe and aimed to unravel the underlying causes [13].

One of the publications from the EQUAL study on elderly patients with advanced CKD disclosed clinically relevant physiological accelerations in patient trajectories that started ∼6 to 12 months prior to death [14]. These findings can help to better inform patients and families about expectations and (end-of-life) care, and for setting up clinical alert systems. Another EQUAL study showed an 18% lower risk of first major adverse cardiovascular events in women than in men, and may therefore add to the understanding of gender disparities [15].

The ERA Registry also contributed to a landmark paper estimating that worldwide more than 850 million people have kidney disease [19]. This article makes clear that kidney diseases are among the most common diseases, as this number is twice the estimated number of people with diabetes worldwide. Finally, in this special collection of ERA Registry articles one can also find methodological articles on the external validation of prediction models [20], on inverse probability weighting [21] and on the need for competing risks analysis methods in survival analyses [22].

All in all, 60 years of ERA Registry, and the years to come, are essential for visualizing the burden of KRT as well as the disparities in the epidemiology of KRT. Renal registries can therefore play an important role not only in healthcare planning but also in quality improvement by reducing disparities and optimizing kidney care.
